# Development and characterization of microsatellite markers for *Antennaria corymbosa* (Asteraceae) and close relatives

**DOI:** 10.1002/aps3.11268

**Published:** 2019-06-11

**Authors:** Ramhari Thapa, Randall J. Bayer, Jennifer R. Mandel

**Affiliations:** ^1^ Department of Biological Sciences University of Memphis Memphis Tennessee 38152 USA

**Keywords:** *Antennaria corymbosa*, Asteraceae, genetic diversity, microsatellites, polyploid agamic complexes, population genetics

## Abstract

**Premise:**

The genus *Antennaria* has a complex evolutionary history due to dioecism, excessive polyploidy, and the evolution of polyploid agamic complexes. We developed microsatellite markers from *A. corymbosa* to investigate genetic diversity and population genetic structure in *Antennaria* species.

**Methods and Results:**

Twenty‐four novel microsatellite markers (16 nuclear and eight chloroplast) were developed from *A. corymbosa* using an enriched genomic library. Ten polymorphic nuclear markers were used to characterize genetic variation in five populations of *A. corymbosa*. One to four alleles were found per locus, and the expected heterozygosity and fixation index ranged from 0.00 to 0.675 and −0.033 to 0.610, respectively. We were also able to successfully amplify these markers in five additional *Antennaria* species.

**Conclusions:**

These markers are promising tools to study the population genetics of sexual *Antennaria* species and to investigate interspecific gene flow, clonal diversity, and parentage of *Antennaria* polyploid agamic complexes.

*Antennaria* Gaertn. species are dioecious perennial herbs distributed mainly in the Holarctic region with the major center of diversity in the Rocky Mountains of western North America (Bayer and Chandler, 2007). The genus comprises 32 known sexual diploid/tetraploid species and several polyploid agamic complexes that mostly reproduce by forming asexual seeds (Bayer, 1990). Although the sexual species are morphologically distinct, polyploid agamic complexes show morphological similarities among themselves due to intense hybridization and introgression from shared sexual progenitors (Bayer, 1990; Bayer and Chandler, 2007). Because of their polymorphism, codominant nature, high cross‐transferability rate, and cost effectiveness, microsatellite loci have been used extensively in the study of gene flow, genetic diversity, and parentage analysis (Kalia et al., 2011); however, microsatellite markers have not yet been developed for *Antennaria*.

We developed and characterized 16 nuclear and eight chloroplast microsatellite markers for *A. corymbosa* E. E. Nelson and demonstrated transferability to other species within the genus. These markers will be helpful for population genetic studies of the sexual *Antennaria* species, as well as studies of clonal diversity and parentage analysis of polyploid agamic complexes.

## METHODS AND RESULTS

Total genomic DNA from *A. corymbosa* (Appendix 1) was extracted using the cetyltrimethylammonium bromide (CTAB) method (Doyle and Doyle, 1987) and quantified using a Qubit BR Assay Kit (Life Technologies, Carlsbad, California, USA). A genomic library was prepared with the NEBNext Ultra II DNA Library Prep Kit (New England Biolabs, Ipswich, Massachusetts, USA) following the manufacturer's protocol. Target enrichment was performed using the myBaits COS Compositae/Asteraceae 1Kv1 kit (Arbor Biosciences, Ann Arbor, Michigan, USA) that targets a conserved ortholog set (COS) of loci in Compositae (Mandel et al., 2014). Sequencing was performed on an Illumina MiSeq sequencer (paired end, sequencing chemistry V2, 150 bp; Illumina, San Diego, California, USA) at the Feinstone Center for Genomic Research (University of Memphis, Memphis, Tennessee, USA). Raw sequence files have been deposited to the National Center for Biotechnology Information (NCBI) Sequence Read Archive (BioProject PRJNA514045). We followed the bioinformatics workflow of Mandel et al. (2014) except we used the program SPAdes v. 3.5 (Bankevich et al., 2012) with *k*‐mer values 99, 111, 115, and 127 for the de novo assembly of 2,060,492 quality reads. The 33,457 retained contigs were matched to the COS loci using the PHYLUCE pipeline v.0.1.0 (Faircloth et al., 2012) following the workflow of Mandel et al. (2014). A perfect motif search for microsatellite loci (di‐ to hexanucleotide repeats, minimum length of 12 bp) was carried out using Phobos version 3.3.11 (Mayer, 2006–2010) as implemented in Geneious version R7.1.9 (Kearse et al., 2012). Primers were developed for product sizes ranging 150–450 bp using Primer3 (Untergasser et al., 2012) with default parameters.

Altogether, 19 primer pairs from the contigs matching the COS loci and 22 primers from the unmatched contigs were developed. Similarly, nine chloroplast microsatellite markers were developed from the partial chloroplast genome of *A. corymbosa* assembled by mapping off‐target reads to the *Artemisia frigida* Willd. chloroplast genome (NC_020607). Primers were synthesized at Integrated DNA Technologies (Coralville, Illinois, USA). Amplification of the 50 simple sequence repeat (SSR) loci was first tested in 12 individuals of *A. corymbosa* (Appendix 1). Genomic DNA was extracted from silica gel–dried leaves or herbarium specimens using the E.Z.N.A. SQ Plant DNA Kit (Omega Bio‐Tek, Norcross, Georgia, USA) and purified with the E.Z.N.A. Cycle Pure Kit (Omega Bio‐Tek). PCR was carried out in 15‐μL single‐plex reactions that contained 1.5 μL 10× buffer, 0.5 μL MgCl_2_ (25 nM), 0.2 μL dNTPs (20 nM), 0.35 μL forward primer (5 nM), 0.35 μL reverse primer (20 nM), 0.35 μL unlabeled M13 (CACGACGTTGTAAAACGAC, 10 nM), 0.7 μL *Taq*, and 1.5 μL DNA. A touchdown PCR protocol was used with the following thermal cycler conditions: 95°C initial denaturation for 3 min, 10 cycles of PCR to bolster primer annealing (30 s at 94°C, 30 s annealing temperature decreasing by 1°C from 65°C to 55°C with each cycle, and 60 s at 72°C), followed by 30 cycles of amplification (30 s at 94°C, 30 s at 55°C, and 60 s at 72°C), and a final extension at 72°C for 10 min. PCR products were visualized on 1% agarose gels.

A total of 36 of the 50 primer pairs, including 27 nuclear and nine chloroplast primer pairs, were selected for genotyping. These 36 loci were re‐amplified separately by replacing unlabeled M13 with M13 containing VIC, NED, FAM, or PET fluorophores in the PCR master mix (Table 1). Amplified products were pooled into dilution plates (up to four loci, 5 μL each plus 10 μL water in 30 μL total volume/sample/well). To prepare the run plate for genotyping, 1 μL of the pooled sample was combined with 6.5 μL of GeneScan 500 LIZ Size Standard (Thermo Fisher Scientific, Carlsbad, California, USA). GeneScan 500 LIZ Size Standard was prepared by diluting in formamide (200 μL of GeneScan 500 LIZ Size Standard and 7.4 mL formamide). Genotyping was carried out via capillary electrophoresis of the PCR products on an ABI 3130XL DNA Analyzer (Life Technologies) at the Molecular Resource Center (University of Tennessee, Memphis, Tennessee, USA). The electropherograms were analyzed with GeneMarker version 2.6.3 (SoftGenetics LLC, State College, Pennsylvania, USA). Of the 27 nuclear markers, 10 were polymorphic, six were monomorphic, and 11 showed ambiguous genotypes. Among the nine chloroplast loci, two showed very low variability, six were monomorphic, and one was ambiguous. All polymorphic and monomorphic loci are listed in Table 1.

**Table 1:** Characteristics of developed microsatellite markers for *Antennaria corymbosa*.^a^

Locus	Primer sequences (5′–3′)	Repeat motif	Allele size range (bp)	Fluorescent dye	GenBank accession no.
**Nuclear markers**					
AcSSR655	F: ACGCACCATTAACTCTCCCG	(ATT)_3_	197–212	VIC	MK243474
	R: CGGTGTACGTGATCCTGGAG				
AcSSR652	F: TGGTTGAGTATTGTGGAAGTAACAC	(CGGGTT)_4_	242–260	VIC	MK243473
	R: TGCTACCTTCCTAAAACCGCT				
AcSSR898	F: AGGAGTCTAAGCTTAAGTTTGTGT	(TGG)_3_	211–223	FAM	MK243476
	R: AGAAGCAAGTTTCTCCAACCCT				
AccSSR208	F: ACTATCAAGATGATGACTTTAGCGA	(TTTG)_4_	281–285	NED	MK243463
	R: GACAGCAAGCCCTCCAAGAA				
AccSSR2209	F: TTCGGGTCAAATACGGGTCG	(TTTA)_4_	197–201	FAM	MK243464
	R: TGGTGTAATCCTGTTGGCCC				
AcSSR93	F: TGCAAGAAACACACAAAACA	(ATC)_4_	172–181	VIC	MK243465
	R: ACGAAGAATCAACCGAAGAT				
AcSSR731	F: AACCCACCTCCTAAAACATC	(ATC)_5_	199–202	NED	MK243475
	R: TAATCCTTTGCACAATCCCA				
AcSSR546	F: TCATGCTTAACCAGGTCAAA	(AACCC)_4_	377–394	NED	MK243472
	R: AAGTAGGTAAAGCAAGTGCA				
AcSSR297	F: TTAACAACCCGCTCATGTT	(AAAT)_4_	187–199	FAM	MK243470
	R: TTAAATCCGTCAGCACAAGA				
AcSSR506	F: CGTGGAGTGATTACGGTATT	(TTGT)_4_	254–271	VIC	MK243471
	R: AAGCATGAAAAACAGCAACA				
AcSSR149	F: GTACAAACGGAGCAAAAGAC	(AAAG)_3_	439	PET	MK243466
	R: GGCAACTTTCATTCTCATGG				
AcSSR170	F: CTTTCTCAAGGAGATGGACC	(AGC)_4_	397	PET	MK243467
	R: TTGTAGTCGCTTACTGACAG				
AcSSR238	F: CGCAATGAGATCACTTCAAG	(AAG)_4_	237	NED	MK243468
	R: TTCCGTTTAGGTATCGGAAC				
AcSSR278	F: TTGCTTGTCCCAAATGGCAC	(TTTGAC)_2_	210	FAM	MK243469
	R: AGGGCTCACATTCCAATCTAAA				
AcSSR969	F: TCCTCATCAACGTTATTGCT	(TA)_6_	281	VIC	MK243477
	R: TTACGACGTATGACAGCATT				
AcSSR1038	F: ATTGATGGGTTCATGCTCTT	(TATT)_3_	207	FAM	MK243477
	R: GCCTGTAAAGTTGTAAGGGA				
**Chloroplast markers**					
AcCpSSR66820	F: TGCTAGAGACATAAACAGTCATGGA	(ATGAG)_3_	337	FAM	MK243458
	R: GGCCTAGCTGTACCTACCGT				
AcCpSSR78370	F: CCGATGGTTCTTACTCAGGGA	(AAT)_5_	210–224	VIC	MK243459
	R: CGGGCCTCTTGAATCCTCTC				
AcCpSSR85580	F: TTTGTTCGATGAGCAGATCCA	(AAT)_12_	305	VIC	MK243460
	R: TCTGGATCCAAAGAACCAGTCA				
AcCpSSR159730	F: ACCCTGTGAATTGTGTGAAAGT	(TGAAT)_3_	239	FAM	MK243462
	R: TCAGGAGGAATTTAATGACAGGACA				
AcCpSSR87080	F: TTTGTATCGCAAAACTACGC	(TTA)_3_	447–451	PET	MK243461
	R: TAAATTTCCGAGGACATGCA				
AcCpSSR36790	F: CATACAACCTTGGCAAGAAC	(AAG)_4_	245	NED	MK243457
	R: TTTTTCAAATCCTGCTGCAG				
AcCpSSR12160	F: CGCCTGTCAATTCAATGAAT	(ATTT)_3_	329	VIC	MK243456
	R: TCGCTTGTATTGTTTGTTGG				
AcCpSSR8110	F: TGCAACTATGATCTATGCCC	(TAA)_4_	350	VIC	MK243455
	R: TTTGGACTGGAATTGACGAA				

a M‐13 (CACGACGTTGTAAAACGAC) is added to the 5′ end of forward primers. Optimal annealing temperature is 55°C.

Genetic diversity within the 10 polymorphic nuclear markers was further tested in 10 individuals for each of five populations of *A. corymbosa* (Appendix 1). These populations were collected from regions representing the major center of diversity for the species in the western part of the United States (Bayer, 2006). We could not collect more than 10 individuals per population because the species produces dense mat‐like structures due to stoloniferous growth, and hence it was difficult to obtain more than 10 distinct patches in a collection area. In addition, we did not collect individuals from very close patches as they could be clones. Evidence for null alleles was not detected by MICRO‐CHECKER version 2.2.3 (van Oosterhout et al., 2004). Linkage disequilibrium among alleles of the loci in populations was not calculated due to small population sizes. Genetic diversity parameters including the number of alleles, observed heterozygosity, expected heterozygosity, and fixation index were calculated using GenAlEx v. 6.502 (Peakall and Smouse, 2006). Hardy–Weinberg equilibrium for each locus was calculated using GENEPOP v. 4.2 (Rousset, 2008) (Table 2). Among the five populations of *A. corymbosa* (50 genotyped individuals), the number of alleles per locus ranged from one to four. Similarly, the overall observed heterozygosity, expected heterozygosity, and the fixation index ranged from 0.00 to 1.00, 0.00 to 0.675, and −1.000 to 0.610, respectively. After a Bonferroni correction for multiple testing, with the adjusted *P* value of 0.001, no significant deviations from Hardy–Weinberg equilibrium were detected (Table 2).

**Table 2:** Genetic characterization of 10 polymorphic microsatellite loci for populations of *Antennaria corymbosa*.^a^^,^ b

Locus	cory_3 (*N* = 10)					cory_30 (*N* = 10)					cory_43 (*N* = 10)					cory_45 (*N* = 10)					cory_50 (*N* = 10)				
	*A*	*H*_o_	*H*_e_	*F*	HWE	*A*	*H*_o_	*H*_e_	*F*	HWE	*A*	*H*_o_	*H*_e_	*F*	HWE	*A*	*H*_o_	*H*_e_	*F*	HWE	*A*	*H*_o_	*H*_e_	*F*	HWE
AccSSR208	2	0.500	0.375	−0.333	NA	1	0.000	0.000	NA	NA	2	0.250	0.219	−0.143	NA	1	0.000	0.000	NA	NA	1	0.000	0.000	NA	NA
AccSSR2209	2	1.000	0.500	−1.000	0.010	2	0.889	0.494	−0.800	0.053	2	0.800	0.480	−0.667	0.173	2	0.900	0.495	−0.818	0.045	2	0.700	0.455	−0.538	0.220
AcSSR93	2	0.100	0.095	−0.053	NA	1	0.000	0.000	NA	NA	2	0.556	0.401	−0.385	1.000	2	0.300	0.255	−0.176	1.000	2	0.200	0.180	−0.111	1.000
AcSSR297	2	0.100	0.095	−0.053	NA	2	0.100	0.095	−0.053	NA	3	0.500	0.505	0.010	0.077	3	0.300	0.265	−0.132	1.000	2	0.100	0.095	−0.053	NA
AcSSR506	1	0.000	0.000	NA	NA	3	0.400	0.335	−0.194	1.000	2	0.700	0.455	−0.538	0.220	2	0.600	0.420	−0.429	0.484	2	0.500	0.375	−0.333	1.000
AcSSR546	3	0.125	0.320	0.610	0.068	2	0.714	0.459	−0.556	0.438	2	0.200	0.180	−0.111	1.000	2	0.300	0.255	−0.176	1.000	1	0.000	0.000	NA	NA
AcSSR652	3	0.778	0.648	−0.200	0.750	3	0.600	0.635	0.055	0.469	4	0.600	0.675	0.111	0.366	2	0.200	0.420	0.524	0.133	2	0.800	0.480	−0.667	0.175
AcSSR655	2	0.300	0.455	0.341	0.483	2	0.200	0.480	0.583	0.079	2	0.200	0.320	0.375	0.304	4	0.900	0.625	−0.440	0.151	2	0.600	0.480	−0.250	1.000
AcSSR731	2	0.889	0.494	−0.800	0.054	2	0.900	0.495	−0.818	0.055	2	1.000	0.500	−1.000	0.007	2	1.000	0.500	−1.000	0.007	2	1.000	0.500	−1.000	0.007
AcSSR898	2	0.100	0.095	−0.053	NA	1	0.000	0.000	NA	NA	2	0.200	0.180	−0.111	1.000	1	0.000	0.000	NA	NA	1	0.000	0.000	NA	NA

Note

*A* = number of alleles; *F* = fixation index; *H*_e_ = expected heterozygosity; *H*_o_ = observed heterozygosity; HWE = Hardy–Weinberg equilibrium; *N* = number of individuals sampled; NA = not applicable.

a Loci were pooled for genotyping in the following manner: (AccSSR208, AcSSR93), (AcSSR546, AccSSR2209, AcSSR652, AcSSR731), and (AcSSR297, AcSSR506, AcSSR655, AcSSR898).

b Locality and voucher information are provided in Appendix 1.

Cross‐species amplification of the markers was also tested in five species of *Antennaria* including *A. microphylla* Rydb., *A. pulchella* Greene, *A. racemosa* Hook., *A. rosulata* Rydb., and *A. umbrinella* Rydb. (Appendix 1). These taxa were chosen as they all occur in the Catipes clade and are potential hybridizers (Bayer, 1990; Bayer and Chandler, 2007). Among 10 polymorphic nuclear markers in *A. corymbosa*, locus AcSSR506 was monomorphic in all species except *A. racemosa*, and locus AcSSR655 was monomorphic in *A. pulchella* and *A. rosulata*. As in *A. corymbosa*, six nuclear loci were monomorphic in all five species. Among the eight chloroplast markers, loci AcCpSSR8110 and AcCpSSR87080 were polymorphic in *A. racemosa*, and locus AcCpSSR85580 was polymorphic in *A. umbrinella* (Table 3). To assess the ability of the markers for resolving relationships at the species level, we produced a NeighborNet split network using SplitsTree4 v. 4.14.5 (Huson and Bryant, 2006) and undertook principal coordinate analysis (PCoA) using GenAlEx for 12 randomly sampled individuals from each of the six *Antennaria* species (Appendix S1). The six *Antennaria* species were well resolved in the SplitsTree network, whereas in the PCoA, *A. corymbosa*,* A. umbrinella*, and *A. rosulata* were not as genetically distinguishable.

**Table 3:** Cross‐species amplification of the 23 microsatellite markers developed for *Antennaria corymbosa* in five additional *Antennaria* species.^a^

Loci	*A. microphylla* (*N* = 12)			*A. pulchella* (*N* = 12)			*A. racemosa* (*N* = 12)			*A. rosulata* (*N* = 12)			*A. umbrinella* (*N* = 12)		
	Success	*A*	Allele size range (bp)	Success	*A*	Allele size range (bp)	Success	*A*	Allele size range (bp)	Success	*A*	Allele size range (bp)	Success	*A*	Allele size range (bp)
**Nuclear markers (polymorphic)**															
AccSSR208	***	3	271–285	**	3	271–289	**	7	279–301	**	3	279–285	**	3	271–285
AcSSR93	***	2	172–181	**	4	172–189	**	3	172–189	**	3	172–181	**	4	171–285
AccSSR2209	***	2	197–200	*	3	197–202	**	4	197–207	***	2	197–200	**	2	197–200
AcSSR546	**	3	377–388	*	2	387–392	**	5	382–392	***	6	388–398	*	4	382–397
AcSSR731	**	2	199–202	**	2	199–202	***	2	199–202	***	2	199–202	**	3	199–211
AcSSR652	***	3	242–254	***	4	242–260	***	2	242–254	***	4	242–260	***	3	242–254
AcSSR655	***	3	202–212	**	1	199	***	2	199–202	***	1	199	***	5	197–210
AcSSR506	***	1	267	*	1	267	***	4	259–275	***	1	267	***	1	267
AcSSR297	***	2	195–199	**	3	187–204	***	2	199–204	***	2	195–199	**	3	195–204
AcSSR898	**	2	211–223	**	3	208–213	**	3	211–226	**	2	211–223	**	2	211–223
**Nuclear markers (monomorphic)**															
AcSSR149	***	1	439	***	1	439	***	1	439	***	1	439	***	1	439
AcSSR170	***	1	397	***	1	397	***	1	397	***	1	397	***	1	397
AcSSR238	***	1	237	***	1	237	***	1	237	***	1	237	***	1	237
AcSSR278	**	1	210	**	1	210	**	1	210	***	1	210	***	1	210
AcSSR1038	***	1	207	***	1	207	***	1	207	***	1	207	***	1	207
AcSSR969	***	1	301	***	1	301	***	1	301	***	1	301	***	1	301
**Chloroplast markers (polymorphic, monomorphic)**															
AcCpSSR8110	***	1	350	***	1	350	***	2	350–356	***	1	350	***	1	350
AcCpSSR12160	***	1	329	***	1	329	***	1	329	***	1	329	***	1	329
AcCpSSR36790	***	1	245	***	1	245	***	1	245	***	1	245	***	1	245
AcCpSSR66820	***	1	337	***	1	337	***	1	337	***	1	337	***	1	337
AcCpSSR78370	***	1	224	***	1	224	***	1	224	***	1	224	***	1	224
AcCpSSR85580	***	1	305	***	1	305	***	1	305	***	1	305	***	2	294–305
AcCpSSR87080	***	1	451	***	1	451	***	2	451–455	***	1	451	***	1	451
AcCpSSR159730	***	1	239	***	1	239	***	1	239	***	1	239	***	1	239

Note

*A* = number of alleles; *N* = number of samples; * = amplification and scoring <50%; ** = amplification and scoring >50% but <90%; *** = amplification and scoring >90%.

^a^Locality and voucher information are provided in Appendix 1.

## Conclusions

The novel microsatellite markers described in this study are the first developed in the genus *Antennaria*. These markers will be helpful for the future population genetic studies of widely distributed *A. corymbosa* populations. With a high cross‐species amplification rate, these markers will be useful in the study of hybridization, interspecific gene flow, and parentage analysis of other *Antennaria* diploid species and polyploid agamic complexes.

## Supporting information


**APPENDIX S1.** (A) NeighborNet split network for six species of *Antennaria*. Numbers indicate population number followed by letters for the individual identification number in a population. (B) Principal coordinate analysis for the six *Antennaria* species based on 10 polymorphic microsatellite markers.

## Data Availability

Raw sequencing reads were deposited in the National Center for Biotechnology Information (NCBI) Sequence Read Archive (BioProject PRJNA514045). Sequence information for the developed primers has been deposited in NCBI's GenBank, and accession numbers are provided in Table 1.

## Voucher information for *Antennaria* species used in this study.

**Table:**

Species	*N*	Collection no./Year/Collectors^a^	Voucher location^b^	Collection locality	Geographic coordinates
*A. corymbosa* E. E. Nelson^c^	1	M‐508/1985/R.J. Bayer & Lebedyk	ALTA	Beaverhead Co., MT, USA	45°13′48.0″N, 111°27′00.0″W
*A. corymbosa* d	10	WY‐04001/2004/R.J. Bayer, G. Chandler & B. Robertson	MEM	Sheridan Co., WY, USA	44°25′47.4″N, 107°45′22.9″W
*A. corymbosa* d	10	MT‐04028/2004/R.J. Bayer, G. Chandler & B. Robertson	MEM	Granite Co., MT, USA	46°13′26.8″N, 113°14′54.9″W
*A. corymbosa* d	10	CO‐07001/2007/R.J. Bayer, G. Chandler, B. Robertson, C. Blanchfield & P. Kemmis	MEM	Gunnison Co., CO, USA	39°01′25.6″N, 107°03′09.3″W
*A. corymbosa* d	10	CO‐07003/2007/R.J. Bayer, G. Chandler, B. Robertson, C. Blanchfield & P. Kemmis	MEM	Gunnison Co., CO, USA	38°50′03.6″N, 106°25′05.8″W
*A. corymbosa* d	10	CO‐07008/2007/R.J. Bayer, G. Chandler, B. Robertson, C. Blanchfield & P. Kemmis	MEM	Gunnison Co., CO, USA	38°49′60.0″N, 107°06′00.0″W
*A*. *microphylla* Rydb.^d^	4	CO‐07004/2007/R.J. Bayer, G. Chandler, B. Robertson, C. Blanchfield & P. Kemmis	MEM	Gunnison Co., CO, USA	38°50′03.6″N, 106°25′05.8″W
*A. microphylla* d	4	CO‐070011/2007/R.J. Bayer, G. Chandler, B. Robertson, C. Blanchfield & P. Kemmis	MEM	Saguache Co., CO, USA	37°59′60.0″N, 106°00′00.0″W
*A. microphylla* d	4	CO‐17002/2017/R.J. Bayer, R. Thapa, N.P. Prather & S. M. Ballou	MEM	Conejos Co., CO, USA	37°09′01.0″N, 106°24′58.2″W
*A. pulchella* Greene^e^	4	CA‐720/1987/R.J. Bayer, R. Deluca & D. Lebedyk	ALTA	Mono Co., CA, USA	37°57′00.0″N, 119°18′00.0″W
*A. pulchella* e	4	CA‐724/1987/R.J. Bayer, R. Deluca & D. Lebedyk	ALTA	Inyo Co., CA, USA	37°10′60.0″N, 118°38′00.0″W
*A. pulchella* e	4	CA‐732/1987/R.J. Bayer, R. Deluca & D. Lebedyk	ALTA	Inyo Co., CA, USA	37°07′60.0″N, 118°34′00.0″W
*A. racemosa* Hook.^d^	4	MT‐04006/2004/R.J. Bayer, G. Chandler & B. Robertson	MEM	Gallatin Co., MT, USA	45°53′30.1″N, 110°53′39.6″W
*A. racemosa* d	4	MT‐04015/2004/R.J. Bayer, G. Chandler & B. Robertson	MEM	Flathead Co., MT, USA	48°13′08.9″N, 113°19′43.5″W
*A. racemosa* d	4	MT‐04022/2004/R.J. Bayer, G. Chandler & B. Robertson	MEM	Deer Lodge Co., MT, USA	46°14′21.9″N, 112°35′08.4″W
*A. rosulata* Rydb.^d^	4	NM‐17002/2017/R.J. Bayer, R. Thapa, N.P. Prather & S. M. Ballou	MEM	Rio Arriba Co., NM, USA	36°39′17.8″N, 106°02′30.3″W
*A. rosulata* d	4	CO‐17005/2017/R.J. Bayer, R. Thapa, N.P. Prather & S. M. Ballou	MEM	Saguache Co., CO, USA	38°13′13.9″N, 106°36′25.4″W
*A. rosulata* d	4	AZ‐17010/2017/R.J. Bayer, R. Thapa, N.P. Prather & S. M. Ballou	MEM	Coconino Co., AZ, USA	34°59′10.4″N, 111°27′35.5″W
*A. umbrinella* Rydb.^d^	4	WY‐04004/2004/R.J. Bayer, G. Chandler & B. Robertson	MEM	Big Horn Co., WY, USA	44°48′18.9″N, 107°53′34.9″W
*A. umbrinella* d	4	MT‐04003/2004/ R.J. Bayer, G. Chandler & B. Robertson	MEM	Gallatin Co., MT, USA	45°05′35.4″N, 111°12′29.1″W
*A. umbrinella* d	4	MT‐04027/2004/R.J. Bayer, G. Chandler & B. Robertson	MEM	Madison Co., MT, USA	45°04′29.4″N, 111°52′04.9″W

Note

*N* = number of individuals sampled.

a The five *A. corymbosa* collections served as the five populations described in Table 2 and correspond to the collection numbers as follows: cory_3 = WY‐04001, cory_30 = MT‐04028, cory_43 = CO‐07001, cory_45 = CO‐07003, and cory_50 = CO‐07008.

b Standard herbarium acronyms following Index Herbariorum (http://sweetgum.nybg.org/science/ih/).

c Specimen used for next‐generation sequencing.

d DNA from silica gel–dried leaves.

e DNA from herbarium specimens.
