# Supplementary material for: Development and characterization of microsatellite markers for Antennaria corymbosa (Asteraceae) and close relatives
Source: Appl Plant Sci. 2019 Jun 11;7(6):e11268. doi: 10.1002/aps3.11268 (PMC6580985; doi:10.1002/aps3.11268)

APPENDIX S1. (A) NeighborNet split network for six species of *Antennaria*. Numbers indicate population number followed by letters for the individual identification number in a population. (B) Principal coordinate analysis for the six *Antennaria* species based on 10 polymorphic microsatellite markers.

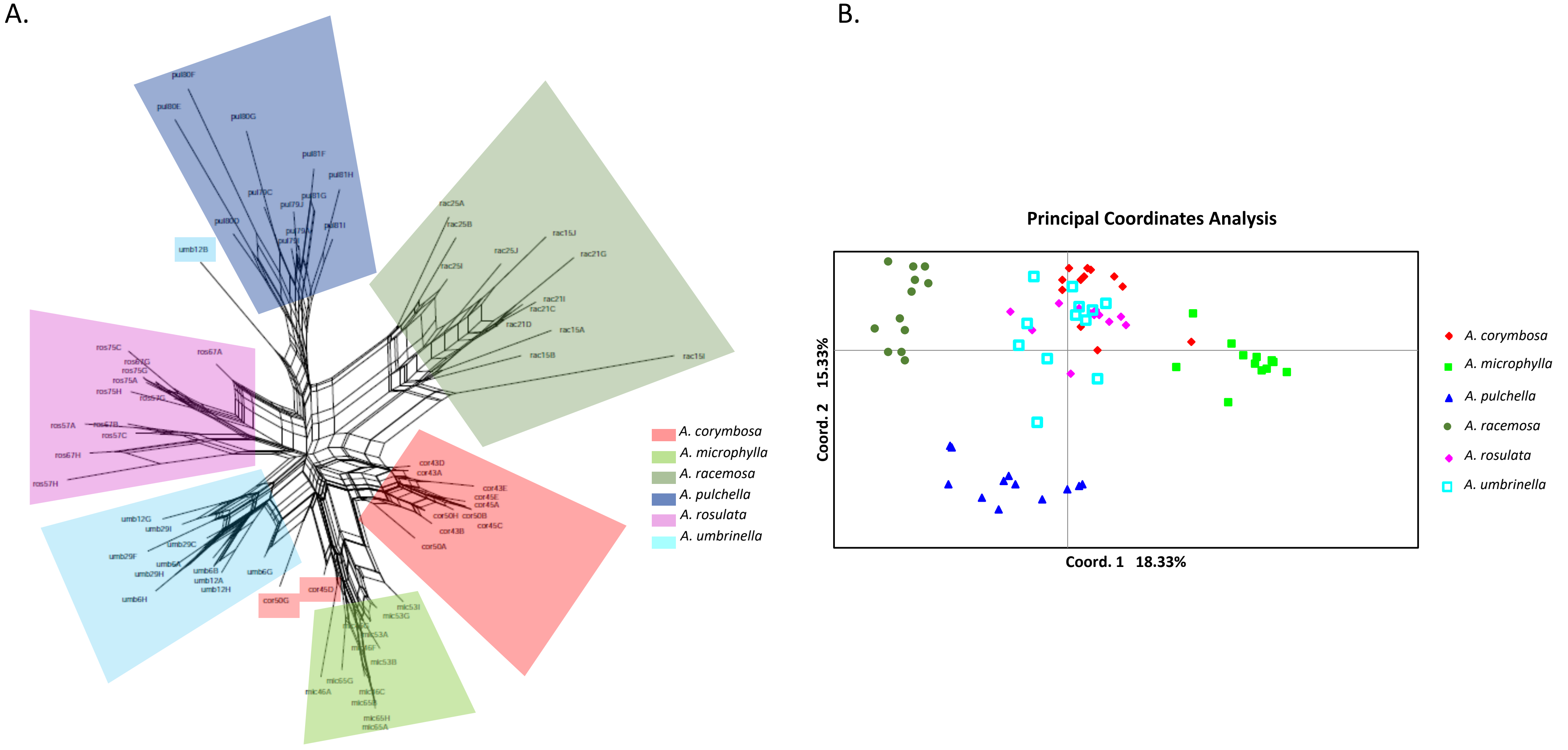

Supplement: Supplementary file 1 — APPENDIX S1. (A) NeighborNet split network for six species of Antennaria. Numbers indicate population number followed by letters for the individual identification number in a population. (B) Principal coordinate analysis for the six Antennaria species based on 10 polymorphic microsatellite markers. [file APS3-7-e11268-s001.pdf]
